# Impact of vitamins A, B, C, D, and E supplementation on improvement and mortality rate in ICU patients with coronavirus-19: a structured summary of a study protocol for a randomized controlled trial

**DOI:** 10.1186/s13063-020-04547-0

**Published:** 2020-07-06

**Authors:** Mohammad Taghi Beigmohammadi, Sama Bitarafan, Azin Hoseindokht, Alireza Abdollahi, Laya Amoozadeh, Maedeh Mahmoodi Ali Abadi, Morteza Foroumandi

**Affiliations:** 1 Anaesthesiology and Intensive Care Department, Imam Khomeini Hospital Complex, Tehran University of Medical Sciences, Tehran, Iran; 2 Iranian Center of Neurological Research (ICNR), Neuroscience Institute, Imam Khomeini Hospital Complex, Tehran University of Medical Sciences, Keshavarz Blvd, Tehran, 1419733141 Iran; 3 Department of Pathology, School of Medicine, Imam Khomeini Hospital Complex, Tehran University of Medical Sciences, Tehran, Iran; 4 Breast Disease Research Center (BDRC), Tehran University of Medical Sciences, Tehran, Iran; 5 Department of Laboratory, Imam Khomeini Hospital Complex, Tehran University of Medical Sciences, Tehran, Iran

**Keywords:** COVID-19, Randomized controlled trial, Protocol, Vitamin B, Vitamin A, Vitamin D, Vitamin E, Vitamin C, Supplementation, Mortality rate, Intensive care unit

## Abstract

**Objectives:**

This study will evaluate the main hypothesis that supplementation with vitamins A, B, C, D, and E significantly improves the severity and mortality rate in ICU patients with COVID-19.

**Trial design:**

This study is a randomized, single-blinded, two-arm (1:1 ratio) parallel group clinical trial.

**Participants:**

We are conducting this study in patients with COVID-19 admitted to intensive care units at the Imam Khomeini Hospital Complex in Tehran, Iran.

The inclusion criteria are as follows: (1) aged between 20 and 60 years, (2) both male and female patients with COVID-19, (3) clinical or definitive diagnosis (using polymerase chain reaction (PCR) test), (4) patients have not participated in other clinical trials, and (5) no renal or hepatic abnormalities.

The exclusion criteria are as follows: (1) patients with specific and rare viral diseases such as HIV and (2) patients who have been undergoing chemotherapy for the past month.

**Intervention and comparator:**

Duration of intervention: 7 days from randomization

Intervention in the treatment group:

- Vitamin A 25,000 IU daily
- Vitamin D 600,000 IU once during study
- Vitamin E 300 IU twice daily
- Vitamin C is taken four times per day
- B vitamins are taken as a daily Soluvit [which included thiamine nitrate 3.1 mg, sodium riboflavin phosphate 4.9 mg (corresponding to vitamin B_2_ 3.6 mg), nicotinamide 40 mg, pyridoxine hydrochloride 4.9 mg (corresponding to vitamin B_6_ 4.0 mg), sodium pantothenate 16.5 mg (corresponding to pantothenic acid 15 mg), sodium ascorbate 113 mg (corresponding to vitamin C 100 mg), biotin 60 μg, folic acid 400 μg, and cyanocobalamin 5 μg]

The control group will not receive any supplements or placebo.

All supplements are made in Iran except for Soluvit (from Fresenius Kabi, New Zealand).

**Main outcomes:**

1. Weight, height, and BMI
2. Severity of pulmonary involvement according to CT scan
3. Respiratory support (invasive or non-invasive)
4. Percentage of oxygen saturation (SpO2 level)
5. Serum levels of WBC, CRP, ESR, IL6, IFN-G, and TNF-α
6. The patient’s body temperature
7. The presence or absence of involvement of organs other than the lungs (e.g., heart, liver, kidneys)
8. Duration of hospitalization
9. Mortality rate

**Randomization:**

At baseline, eligible patients were randomly assigned to a 1:1 ratio to one of two groups: intervention and control. Block randomization is used based on the gender of patients.

**Blinding (masking):**

Patients are unaware of being placed in the intervention or control groups after signing consent. All treatment staff will be aware of which group each of the patients is in due to the specific conditions of the ICU and the absence of placebo for the control group.

**Numbers to be randomized (sample size):**

The researchers plan to include 60 patients in total, with 30 patients in each group.

**Trial status:**

This is the first version of the protocol which started on April 2, 2020. Recruitment began April 2, 2020, and is expected to be complete by July 4, 2020.

**Trial registration:**

The Iranian Registry of Clinical Trials IRCT20200319046819N1. Registered on April 4, 2020

**Full protocol:**

The full protocol is attached as an additional file, accessible from the *Trials* website (Additional file 1). In the interest in expediting dissemination of this material, the familiar formatting has been eliminated; this letter serves as a summary of the key elements of the full protocol (Fig. 1, Table 1).

**Table 1:** Outcome assessment

Level	Outcomes	Measurement
**Primary**	WBC, CRP, IL-6, TNF-α, IFN-G ESR	Laboratory blood test
	Intensity of pulmonary involvement	CT scan
	Mortality rate	Observation
**Secondary**	BMI	According to weight and height
	Duration of hospitalization	Observation
	Saturation percentage of blood oxygen	Pulse oximeter

**Fig. 1:**
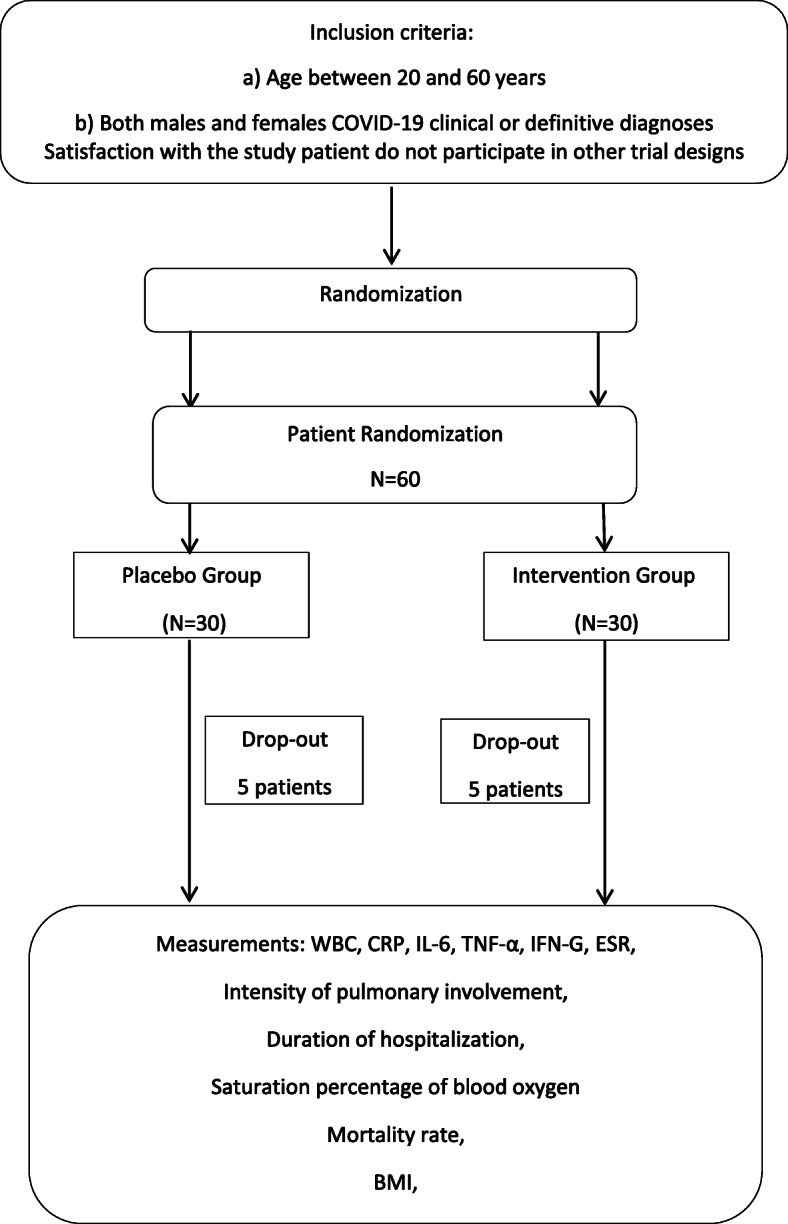
Summary of patient flow diagram

## Supplementary information

**Additional file 1.**

## Data Availability

The corresponding and first authors have access to the data in a SPSS file.

