# Supplementary material for: Impact of vitamins A, B, C, D, and E supplementation on improvement and mortality rate in ICU patients with coronavirus-19: a structured summary of a study protocol for a randomized controlled trial
Source: Trials. 2020 Jul 6;21:614. doi: 10.1186/s13063-020-04547-0 (PMC7336105; doi:10.1186/s13063-020-04547-0)
Supplement: Supplementary file 1 — Additional file 1. [file 13063_2020_4547_MOESM1_ESM.docx]

**Title:**

"Impact of vitamin A, B, C, D, E supplementation on improvement and mortality rate in patients with Coronavirus-19 admitted in intensive care unit:

Study protocol for a randomized clinical trial"

**Abstract**

**Background:** This study is designed to assess the main hypothesis that supplementation with vitamin A, B, C, D, and E improves the severity and mortality rate in patients with Coronavirus-19 (COVID-19).

**Methods:** This study is a randomized, one blinded, and two-arm parallel clinical trial in intensive care units (ICU) of Imam Khomeini Hospital. Sixty patients with COVID-19 are randomly assigned in two intervention and control groups. Intervention group receives 25000 IU vitamin A, 600000 IU vitamin D, twice daily 300 IU vitamin E, 500 mg vitamin C four times in a day and B vitamin complex (Soluvit) one ampule daily. Control group does not receive any supplements. Our primary outcome comprised WBC, CRP, ESR, IL-6, TNF-α, IFN-G, intensity of pulmonary involvement and mortality rate. As well as our secondary outcome comprised Body Mass Index (BMI), duration of hospitalization and saturation percentage of blood oxygen. The expected parameters will be measured at the baseline and end of the intervention.

**Discussion:** Our study should define whether vitamin A, B, C, D, E, supplementation improves the inflammatory parameters, intensity of pulmonary involvement and mortality rate in patients with COVID-19.

**Trial registration:**

IR.TUMS.VCR.REC.1399.090

**Keywords**

COVID-19, Randomised controlled trial, protocol, Vitamin B, Vitamin A, Vitamin D, Vitamin E, Vitamin C, Supplementation, Mortality rate, Intensive care unit

**Background**

Around the beginning of 2020, a novel coronavirus (COVID-19) was observed in Wuhan, China with a rapid infection rate and potent transmission [1]. Infection with COVID-19 is associated with upper respiratory tract involvement and symptoms included fever, headache, and cough. All epidemiologic studies have shown that viruses emerge unexpectedly, spread easily, and have catastrophic consequences [2]. Because of no definite medical therapy for COVID-19, aggressive treatments and supportive therapies such as supplementation are mainly recommended in severe cases [3].

Nutritional status is an important factor for adolescent immune responses in patients and malnutrition conditions, both under and overweight, are the most common causes of immune dysregulation worldwide. Protein-energy malnutrition (PEM) is associated with significant impairment of cellular mediated immunity, phagocytic function, complement system, secreted immunoglobulin A antibody concentration, and cytokine production. Among the micronutrients that play important roles in the immune response are vitamins A, D, C, E and B complex [4].

In this way, several studies have shown that vitamin A can lead to decrease complications and mortality in various infectious diseases and vitamin C can reduce the rate of pneumonia [5-7]. On the other hand, several studies have reported that vitamins B, D and E deficiency can make the body more vulnerable to the virus and impair the immune system responses [5, 8-10].

Due to the widespread prevalence, severity, mortality rate and unknown effective medical treatment of COVID-19, effective complementary medicines are needed to improve immune system regulation and patient condition. Our study evaluate the effectiveness of a multivitamin supplementation, including vitamins A, B, C, D, E, on improving and mortality rates in patients with COVID-19 admired in intensive care units.

**Methods**

1. **Objectives and design**

We conduct a randomized, one-sided blinded and two-arm clinical trial in ICUs at Imam Khomeini Hospital Complex in Tehran, Iran. This study compares the effect of supplementation with injectable vitamins A, B, C, D, and E on 30 patients with COVID-19 admitted in intensive care units in intervention group versus 30 patients with COVID-19 in control group. This study experiments the main hypothesis that the intervention can significantly improve the health severity and mortality rate in patients. The medical staff cannot be blinded to intervention due to injection of supplementation in ICU and impossibility of create the same conditions for injection of placebo. The study was approved by the ethics committee of the medical university of Tehran on 31 March 2020 (IR.TUMS.VCR.REC.1399.090). This trial was registered at the Iranian registry of clinical trials on 4 April 2020 (IRCT20200319046819N1). The secondary outcome comprises Body Mass Index, duration of hospitalization, saturation percentage of blood oxygen.

1. **Study population**

Sixty patients with COVID-19 admitted in intensive care units of Imam Khomeini Hospital Complex are entered to the study.

Inclusion criteria are: Age between 20 and 60 years, both males and females, clinical or definitive diagnosis (positive RT-PCR or chest CT presentations) for COVID-19, non-intubated patients with moderate illness, satisfaction with the study patient, patients should not participate in other trial studies, lack of renal and hepatic abnormalities,

Exclusion criteria are: Patients with specific and rare viral diseases such as HIV etc, patients have been undergoing chemotherapy for the past month, chronic kidney disease (CKD) or acute kidney disease (AKI) patients, other contraindications in use of supplements.

1. **Intervention**

Treatment group receives supplements for 7 days in injectable form. Supplements are vitamin A 25000 IU daily, vitamin D 600000 IU once during intervention period, vitamin E 300 IU twice daily, 500 mg vitamin C is taken four times per day and finally B vitamins are taken as a daily Soluvit: Thiamine nitrate 3.1 mg, Sodiumriboflavine phosphate 4.9 mg (corresponding to Vitamin B2 3.6mg), Nicotinamide 40 mg, Pyridoxine hydrochloride 4.9 mg (corresponding to Vitamin B6 4.0mg), Sodium pantothenate 16.5 mg(corresponding to Pantothenic acid15 mg), Sodium ascorbate 113 mg (corresponding to Vitamin C 100mg), Biotin 60 μg, Folic acid 400 μg, Cyanocobalamin 5 μg; control group will not receive any supplementation or placebo. Except for Soluvit (Fresenius Kabi New Zealand), all supplements are made in Iran. Control group receives the same medicine but no placebo instead of supplements.

1. **Measurements**

- **General and baseline information**

Clinical signs, demographic data, anthropometric measures, and the presence of comorbidities are recorded as general information from patients with COVID-19. The primary outcomes are the WBC (differences), CRP, ESR, IL-6, TNF-α, and IFN-G as inflammatory status measurement by laboratory blood test. Blood samples will be taken from patients admitted to the ICU at morning. Additional primary outcome comprise intensity of pulmonary involvement and mortality rate as determined by CT scan and observation, respectively. The secondary outcome comprises BMI as calculated by formula [weight / (height).]. Additional secondary outcomes are the duration of hospitalization, saturation percentage of blood oxygen (below to 92% or lower), which is known as a marker for identifying COVID-19 disease. All outcomes measure at the laboratory center of Imam Khomeini Hospital Complex.

- **Follow up**

Participants are followed for the primary and secondary outcomes within study duration. Follow-up is performed by trained individuals with the same condition (Table1).

1. **Data analysis**

Quantitative variables will be reported as average and standard deviation and qualitative variables will be reported as abundance and percentage. Also, to compare different variables in two groups with each other based on whether the distribution of variables is normal or abnormal, appropriate parametric and nonparametric tests will be used.

1. **Sample size consideration**

According to our knowledge, no data on the effect of vitamins A, B, C, D, and E supplements on COVID-19 are not available for sample size calculation. We considered a required minimum sample size of 27 patients to show a 70% estimation of mortality rate in control group versus 30% estimation of mortality rate in intervention group. Therefore, we will consider recruiting 30 patients in each group to compensate for the 15% drop-outs.

1. **Assignment to intervention**

At baseline of trial, eligible patients were randomly assigned to 1: 1 ratio in two groups of intervention and control. Block randomization is used with participants based on gender. Furthermore, the two groups match according to age and underlying disease.

1. **Ethics**

The Participants will complete a written consent prior of including into the study. Information will be kept confidential of all patients and those can withdraw from the study at any time that does not wish to continue.

1. **Blinding of treatment allocation**

Patients are unaware of being placed in the intervention or control group after declaration of consent. All treatment staff is aware of the patients in which group due to the specific conditions of the ICU and the absence of injectable placebo for control group.

**Discussion**

Present study is the first randomized clinical trial in the world to examine the effectiveness of supplementation with injectable vitamins A, B, C, D, and E, on the Intensity of pulmonary involvement, inflammatory parameters and mortality rate in patients with COVID-19 admitted in ICU.

Some Interesting evidences have shown that inflammatory parameters and cytokines are elevated in COVID-19 patients [11, 12]. Therefore, it seems likely that increasing of illness severity due to viral infection might induce the effects of virus-induced cytopathic, which will be reflected by the increased inflammatory factors [13, 14]. Recent studies have demonstrated that supplementation with some vitamins such as A, B, C, D, and E, can protect people against viral infections [15-19]. These vitamins can reduce the intensity of pulmonary involvement due to increased antioxidant status and improved immune system function [20-24].

The underlying mechanism of vitamins A and C supplementation is to regulate immune system responses by inducing balance between inflammatory and anti-inflammatory factors [5, 6, 25-27]. Vitamins B complex can prevent the intensity of pulmonary infection and regulate the immune system function by regulating protein and energy metabolism [10, 28, 29]. In this way, physiologic role of supplementation with vitamins D and E is to ameliorate autoimmunity by modulating immune system function, regulation of AMP-activated protein kinase (AMPK) signaling pathway, and reducing oxidative stress, respectively [30-36].

According to our knowledge so far, there is no data that have shown the effect of simultaneous supplementation with all vitamins on inflammatory factors and mortality rate in patients with COVID-19. Therefore, our data will show the effect of vitamins on immune system function which will be obtained by inflammatory factor measurements. Also, our data will examine the effect of vitamins on the intensity of pulmonary involvement.

**Acknowledgment:** The special COVID-19 grant in our study is supported by Tehran University of Medical Sciences and Health Services (project registration number: 99-1-101-47104).

**Funding:** Project registration number: 99-1-101-47104

**Conflict of Interest Statement:** There is no conflict of interest.

**Authors' contributions:**

MT B m: Inclusion and exclusion criteria checking, Supervision on sampling and randomization, final approval

S B: Conceptualization, Methodology, analysis and interpretation of data, Reviewing and Editing, final approval

A A: Laboratory equipment and tests, supervision

L A: Inclusion and exclusion criteria checking, supplementation, data collection

A H: Sampling, Inclusion and exclusion criteria checking, supplementation, data gathering

M M: Acquisition of blood sample, Laboratory tests

M F: analysis of pulmonary scans

**References**

1. Organization, W.H., Laboratory testing of human suspected cases of novel coronavirus (‎‎‎ nCoV)‎‎‎ infection: interim guidance, 10 January 2020. 2020, World Health Organization.

2. Zhang, L. and Y.J.J.o.m.v. Liu, Potential Interventions for Novel Coronavirus in China: A Systematic Review. Med virolog. 2020.

3. Huang, X., et al., Epidemiology and Clinical Characteristics of COVID-19. Arch Iran Med, 2020. 23: p. 268-271.

4. Chandra, R.K., Nutrition and the immune system: an introduction. Am J Clin Nutr, 1997. 66: p. 460S-463S.

5. Huang, C., et al., Clinical features of patients infected with 2019 novel coronavirus in Wuhan, China. The lancet, 2020. 395: p. 497-506.

6. Nieman, D.C., et al., Influence of vitamin C supplementation on cytokine changes following an ultramarathon. J Interferon Cytokine Res, 2000. 20: p. 1029-1035.

7. Hemilä, H.J.J.o.A.C., Vitamin C and SARS coronavirus. J Antimicrob Chemoth, 2003. 52: p. 1049-1050.

8. Wang, Y., et al., Modulation of immune function and cytokine production by various levels of vitamin E supplementation during murine AIDS. Immunopharmacology, 1995. 29: p. 225-233.

9. Nag, S., et al., Tannic acid and vitamin E loaded PLGA nanoparticles ameliorate hepatic injury in a chronic alcoholic liver damage model via EGFR-AKT-STAT3 pathway. Nanomedicine, 2020. 15: p. 235-257.

10. Mikkelsen, K., et al., The effects of vitamin B on the immune/cytokine network and their involvement in depression. Maturitas, 2017. 96: p. 58-71.

11. Liu, K., et al., Clinical features of COVID-19 in elderly patients: A comparison with young and middle-aged patients. J Infection, 2020.

12. Tabrizi, R., et al., High prevalence of vitamin d deficiency among iranian population: A systematic review and meta-analysis. Iran J Med Sci, 2018. 43: p. 125.

13. Min, C.-K., et al., Comparative and kinetic analysis of viral shedding and immunological responses in MERS patients representing a broad spectrum of disease severity. Sci Rep, 2016. 6: p. 1-12.

14. Channappanavar, R. and S. Perlman. Pathogenic human coronavirus infections: causes and consequences of cytokine storm and immunopathology. in Seminars in immunopathology. 2017. Springer.

15. Hansdottir, S., et al., Vitamin D decreases respiratory syncytial virus induction of NF-κB–linked chemokines and cytokines in airway epithelium while maintaining the antiviral state. J Immunol, 2010. 184: p. 965-974.

16. Hemilä, H., Vitamin C and infections. Nutrients, 2017. 9: p. 339.

17. Ross, A.C. and C.B. Stephensen, Vitamin A and retinoids in antiviral responses. FASEB J, 1996. 10: p. 979-985.

18. Rehman, Z.U., et al., Supplementation of vitamin e protects chickens from Newcastle disease virus-mediated exacerbation of intestinal oxidative stress and tissue damage. Cell Physiol Biochem, 2018. 47: p. 1655-1666.

19. Mogul, D.B., et al., Development of a Dietary Methyl Donor Food Frequency Questionnaire to Assess Folate and Vitamin B12 Status in Children with Chronic Hepatitis B Virus Infection. Pediatrics, 2018. 203: p. 41-46. e2.

20. Paschalis, V., et al., Low vitamin C values are linked with decreased physical performance and increased oxidative stress: reversal by vitamin C supplementation. Eur J Nutr, 2016. 55: p. 45-53.

21. Jamilian, M., et al., A randomized controlled clinical trial investigating the effects of omega-3 fatty acids and vitamin E co-supplementation on biomarkers of oxidative stress, inflammation and pregnancy outcomes in gestational diabetes. Can J Diabetes, 2017. 41: p. 143-149.

22. Ford, T.C., et al., The effect of a high-dose vitamin b multivitamin supplement on the relationship between brain metabolism and blood biomarkers of oxidative stress: a randomized control trial. Nutrients, 2018. 10: p. 1860.

23. Chirumbolo, S., et al., The role of vitamin D in the immune system as a pro-survival molecule. Clin Ther, 2017. 39: p. 894-916.

24. Mora, J.R., M. Iwata, and U.H. Von Andrian, Vitamin effects on the immune system: vitamins A and D take centre stage. Nat Rev Immunol, 2008. 8: p. 685-698.

25. Hemilä, H., Vitamin C and SARS coronavirus. J Antimicrob Chemoth, 2003. 52: p. 1049-1050.

26. Aukrust, P., et al., Decreased vitamin A levels in common variable immunodeficiency: vitamin A supplementation in vivo enhances immunoglobulin production and downregulates inflammatory responses. Eur J Clin invest, 2000. 30: p. 252-259.

27. Antoniades, C., et al., Vascular endothelium and inflammatory process, in patients with combined Type 2 diabetes mellitus and coronary atherosclerosis: the effects of vitamin C. Diabet Med, 2004. 21: p. 552-558.

28. Depeint, F., et al., Mitochondrial function and toxicity: role of the B vitamin family on mitochondrial energy metabolism. Chem Biol Interact, 2006. 163: p. 94-112.

29. Matte, J., C. Girard, and B. Sève, Effects of long-term parenteral administration of vitamin B 6 on B 6 status and some aspects of the glucose and protein metabolism of early-weaned piglets. Br J Nutr, 2001. 85(1): p. 11-21.

30. Takahashi, K., et al., Human neutrophils express messenger RNA of vitamin D receptor and respond to 1 α, 25-dihydroxyvitamin D3. Immunopharm Immunot, 2002. 24: p. 335-347.

31. Alitalo, A., Human anti-infectious defence may be enhanced by vitamin D. Duodecim; 2010. 126: p. 1127-1134.

32. Hussain, M., et al., Effect of organic and inorganic selenium with and without vitamine E on immune system of broilers. Pak Vet J, 2004. 24: p. 1-4.

33. Paiva, S., et al., Assessment of vitamin A status in chronic obstructive pulmonary disease patients and healthy smokers. Am J Clin Nutr, 1996. 64: p. 928-934.

34. Kim, W.-Y., et al., Combined vitamin C, hydrocortisone, and thiamine therapy for patients with severe pneumonia who were admitted to the intensive care unit: Propensity score-based analysis of a before-after cohort study. J Crit Care, 2018. 47: p. 211-218.

35. Baeke, F., et al., Vitamin D: modulator of the immune system. Curr Opin Pharmacol, 2010. 10: p. 482-496.

36. Schwalfenberg, G.K., A review of the critical role of vitamin D in the functioning of the immune system and the clinical implications of vitamin D deficiency. Mol Nutr Food Res, 2011. 55: p. 96-108.
